# Estimating Workforce-Related Economic Impact of a Pandemic on the Commonwealth of Virginia

**DOI:** 10.1109/TSMCA.2009.2033032

**Published:** 2009-11-06

**Authors:** Mark J. Orsi, Joost R. Santos

**Affiliations:** 1 Center for Risk Management of Engineering SystemsUniversity of Virginia Charlottesville VA 22904-4160 USA; 2 Department of Engineering Management and Systems EngineeringThe George Washington University Washington DC 20052 USA

**Keywords:** Economic interdependence, inoperability input–output modeling, pandemic, resilience, Virginia

## Abstract

A pandemic outbreak is one of the major planning scenarios considered by emergency-preparedness policymakers. The consequences of a pandemic can significantly affect and disrupt a large spectrum of workforce sectors in today's society. This paper, motivated by the impact of a pandemic, extends the formulation of the dynamic inoperability input–output model (DIIM) to account for economic perturbations resulting from such an event, which creates a time-varying and probabilistic inoperability to the workforce. A pandemic is a unique disaster, because the majority of its direct impacts are workforce related and it does not create significant direct impact to infrastructure. In light of this factor, this paper first develops a method of translating unavailable workforce into a measure of economic-sector inoperability. While previous formulations of the DIIM only allowed for the specification of an initial perturbation, this paper incorporates the fact that a pandemic can cause direct effects to the workforce over the recovery period. Given the uncertainty associated with the impact of a pandemic, this paper develops a simulation framework to account for the possible variations in realizations of the pandemic. The enhancements to the DIIM formulation are incorporated into a MatLab program and then applied to a case study to simulate a pandemic scenario in the Commonwealth of Virginia.

## I. Introduction

A Pandemic outbreak is one of the major planning scenarios considered by emergency-preparedness policymakers. According to disease control experts, a pandemic—an infection that spreads widely and affects a significant proportion of the population—is inevitable [1]. Although a pandemic event has not struck the modern society, historical accounts have clearly manifested its plausibility and the dire consequences it can bring to the general population. With the rise of new infections such as the severe acute respiratory syndrome, avian flu, swine flu, as well as the threat of bioterrorism, experts from the General Accounting Office believe it is imminent [2].

A dynamic extension to interdependence modeling is needed to describe the temporal nature of sector recoveries pursuant to a disaster, such as in the case of a pandemic scenario. In particular, we propose an extension to the dynamic inoperability input–output model (DIIM) [3] to model the recovery process for different magnitudes and time-varying disruptions to workforce sectors. Given the initial inoperabilities caused by a disaster, the dynamic extension gives the trajectory of recovery based on the interdependence and resilience characteristics of the industry sectors.

With the looming fear of a pandemic occurring in our present world, it is imperative to develop models for assessing its consequences and for evaluating the efficacy of available mitigation strategies. A pandemic encompasses different consequence categories and can cross multiple geographic jurisdictions. In an attempt to address the complexities inherent in a pandemic (and in any disasters), this paper develops a dynamic and probabilistic model to account for workforce productivity degradations and resulting workforce-induced economic losses. In particular, we use the Commonwealth of Virginia to implement the model and to demonstrate the companion computer tool. The same tool can be used for modeling other regions by customizing the underlying economic and demographic databases.

## II. Supporting Models

In this section, we present summaries of underlying models that support and provide context to the DIIM extension and case study. For brevity and to focus more on the MatLab tool and case-study results, additional details to the supporting models are reviewed in greater detail in a prequel paper [4].

### A. Input–Output Modeling

The Infrastructure Security Partnership describes that understanding interdependencies and the resulting cascading impacts from an impact is essential to develop an effective security plan [5]. The Leontief input–output (I-O) model is a particular method for modeling interdependencies across multiple sectors of a given regional economy [6], [7]. Extensions and current frontiers on I-O analysis can be found in [8]. It is worth noting that the traditional use of I-O analysis for estimating the effects of economic shifts (e.g., changes in consumption) has been extended to other applications such as disaster risk management, environmental impact analysis, and energy consumption, among others. Various studies for estimating losses pursuant to disasters have employed traditional I-O analysis and extended approaches such as computable general equilibrium (CGE) models. Rose and Liao [9] conducted a case study of water-supply disruption scenarios in Portland; they used CGE to account for resilience factors (e.g., substitution and conservation) that business sectors typically consider in order to minimize potential losses. Rose [10] describes that CGE is an extension rather than a replacement of the traditional I-O model. Other disaster-related applications of I-O and CGE models include [11]–[13].

The formulation of the basic Leontief I-O model is shown in (1). The notation ${\bf x}$ refers to the total production output vector. On the other hand, the Leontief technical coefficient matrix ${\bf A}$ represents the ratios of the input of industry $i$ to industry $j$, with respect to the total production requirements of industry $j$. Finally, the notation ${\bf c}$ refers to the final demand vector

$${\bf x} = {\bf Ax} + {\bf c}.\eqno{\hbox{(1)}}$$

### B. Iim

Based on Leontief's work, Haimes and Jiang [14] developed the IIM for interconnected systems. Through matrix manipulations, the IIM introduces the concept of inoperability into the traditional I-O model which augments typical economic loss analysis. The IIM was later expanded by Santos and Haimes [15] to quantify the economic losses triggered by terrorism and other disruptive events to economic systems (or industry sectors). The analysis of economic impacts associated with such events is made possible through the economic I-O data published by the Bureau of Economic Analysis [16]. The formulation of the IIM is as follows:

$${\bf q} = {\bf A}^{\ast}{\bf q} + {\bf c}^{\ast}.\eqno{\hbox{(2)}}$$

In essence, the terms in the formulation in (2) are derived from the Leontief formulation in (1) and are interpreted as follows: ${\bf c}^{\ast}$ is a perturbation vector expressed in terms of normalized degraded final demand (i.e., “as-planned” final demand minus actual final demand, divided by the as-planned production level), ${\bf A}^{\ast}$ is the interdependence matrix which indicates the degree of coupling of the industry sectors, and ${\bf q}$ is the inoperability vector expressed in terms of normalized economic losses.

### C. Diim

The DIIM is an extension of the IIM that provides supplementary risk metrics for assessing the consequences of disasters [17]. The purpose of implementing a dynamic model is to analyze the recovery processes for various sectors in the aftermath of a disaster. Given the initial inoperabilities caused by the disaster, recovery analysis gives the trajectory of recovery based on the interdependence and resilience characteristics of the sectors. In addition to the inoperability and economic loss metrics, the recovery period and resilience metrics generated from the dynamic analysis can provide insights in describing the recovery process for the affected sectors. The model formulation, from Lian and Haimes [3], is as follows:

$${\bf q}(t + 1) = {\bf q}(t) + {\bf K}\left[{\bf A}^{\ast}{\bf q}(t) + {\bf c}^{\ast}(t) - {\bf q}(t)\right].\eqno{\hbox{(3)}}$$

The term ${\bf K}$ is a sector resilience coefficient matrix that represents the rates with which sectors recover to their nominal levels of availability following a disruption. The model dictates that the inoperability level at the following time step ${\bf q}(t + 1)$ is equal to the inoperability at the previous stage ${\bf q}(t)$ plus the effects of the resilience of the sector. As seen in the aforementioned equation, ${\bf K}$ is multiplied with the indirect inoperability resulting from other sectors ${\bf A}^{\ast}{\bf q}(t)$ plus a productivity disruption factor ${\bf c}^{\ast}(t)$ minus the current level of inoperability ${\bf q}(t)$.

While the aforementioned model provides a foundation for measuring the impact of a pandemic, there are certain idiosyncracies associated with such a disaster that are not accounted for in the current formulation. In our DIIM extension, we will explicitly incorporate the analysis and parameterization of disruptions to different workforce sectors.

## III. Probabilistic and Dynamic Extensions

**Fig. 1.**
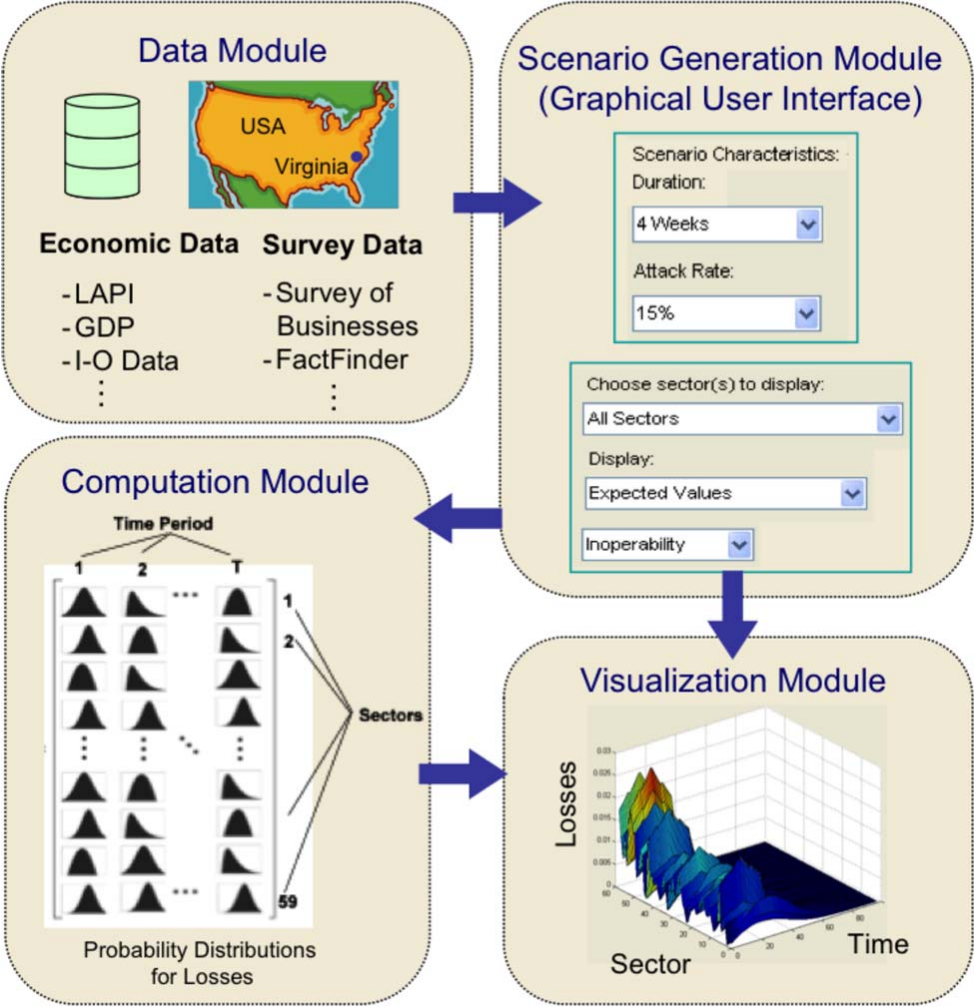
Framework for integrating probabilistic and dynamic extensions with GUI.
2) *Scenario Generation Module*. This module automates the process of entering parameters associated with a given scenario. Sample questions that can be asked (via a pull-down menu in the GUI) are the duration of the scenario and the pandemic attack rates to be simulated. This information will then be fed to the computation module. The user can also specify types of graphs to display with the visualization module.
3) *Computation Module*. This is the computing engine of the program containing the codes for the DIIM application. This module stores the simulation rules and algorithms needed for executing the dynamic and probabilistic extensions. The required scales of computations (time period, sectors, and probability distributions of losses) are also in this module.
4) *Visualization Module*. This shows the results of the simulation based on the GUI-specified inputs. The user has the option to choose from various types of displays such as minimum, expected value, and extreme-event consequences (economic loss and inoperability).

The previous implementation of the DIIM was typically done in a Microsoft Excel workbook. However, with the modifications to the formulation presented in this paper, the Excel workbook is no longer sufficient. This is a result of the need for multidimensional data structures as well as the need for running simulations of these data structures. To better account for this, a MatLab graphical user interface (GUI) was developed to run the simulations and output the results for analysis. Fig. 1 shows a schematic diagram of the MatLab program comprising of four modules: 1) data; 2) scenarios; 3) computation engine; and 4) visualization of results.

1) *Data Module*. This represents a central repository of relevant data pertaining to regional economic and survey data. Examples of economic data include local area personal income, gross domestic product (GDP), and I-O accounts. This module also contains survey data on local businesses and other pertinent sector-specific data.

## IV. Virginia Pandemic Case Study

This section discusses the usage of regional economic data, as well as pandemic data and model results from the Centers for Disease Control and Prevention (CDC) to determine the impact of a pandemic on the economy of the state of Virginia. Results of the model and implications on risk-management decisions are presented and discussed.

This case study focuses on a four-week pandemic scenario in Virginia, as shown in Fig. 1. It explores simulated attack rates, as defined by CDC's FluWorkLoss: 15%, 25%, and 35% [18], [19]. Each attack rate was run with 10 000 simulations. With a run time of roughly 2 s per simulation, this makes the total run time for each simulation about 5.5 h. For brevity, only the details of results for a four-week 15% attack rate will be presented. Nevertheless, comparisons of consequences for other attack rates and durations will be summarized at the end of this section. The different attack rates will be compared in terms of their overall impact, highest impacted sectors, and general behavior.

In subsequent discussions, we will use four different statistical measures for both inoperability and economic loss estimates, namely: 1) minimum; 2) mean; 3) conditional upper tail expectation $f_{4}$; and 4) maximum. The conditional upper tail expectation will be determined as the average of the highest 10% of the simulated values for inoperability and economic loss.

Scenario 1: **Four-Week Pandemic, 15% Attack Rate**

**Fig. 2.**
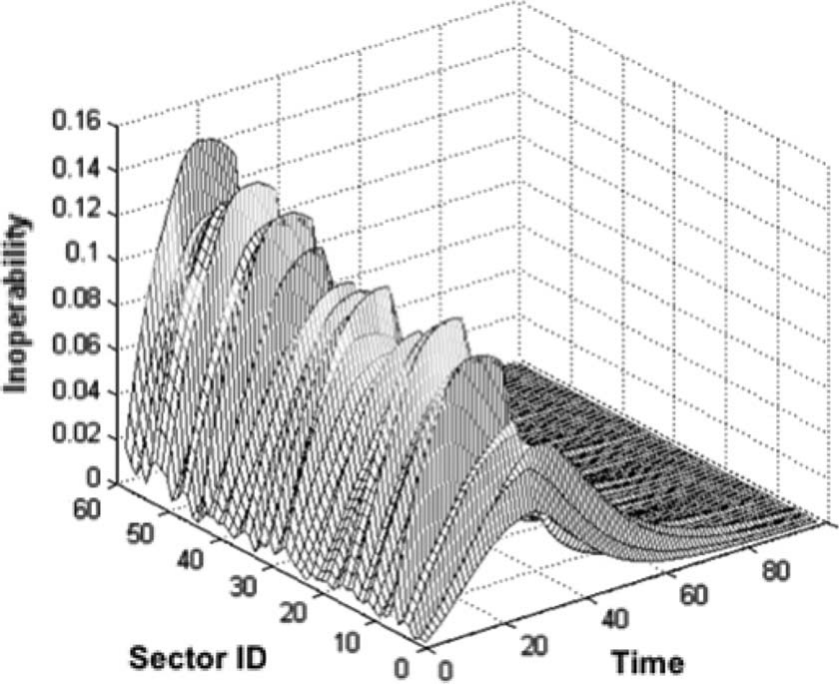
Inoperability for all sectors. Four-week 15%-attack-rate pandemic.

Fig. 2 shows a summary of the inoperability for all sectors over the duration of the pandemic and the time to recover to 1% of maximum inoperability. An interesting behavior of the graph is that the peak inoperability of any sector at any time is around 14%. This means that the 14% inoperability level reflects the growing inoperability in the sector due to sustained levels of absent workforce.

**Fig. 3.**
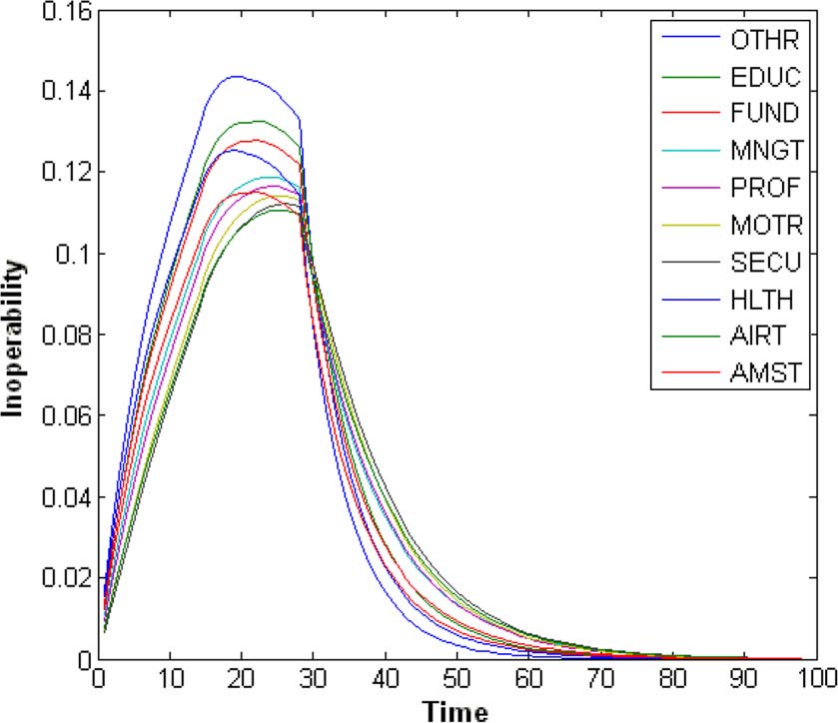
Top ten sectors with highest inoperability values.

Another interesting behavior is the relatively steep recovery beginning at day 28. This is seen more clearly in Fig. 3, a plot of the inoperability of the top ten most inoperable sectors. Right at day 28 (the end of the pandemic), the inoperability begins to recover very rapidly, particularly relative to its previous behavior. These results can again be traced back to the FluWorkLoss inputs, which instantaneously drop from 2% inoperability to zero. Thus, at that point, the model begins to recover as though every person returned to work.

In terms of sectors most impacted by the pandemic, Fig. 3 shows that the top ten sectors in terms of inoperability are the following (in order): 1) other services; 2) educational services; 3) funds, trusts, and other financial vehicles; 4) management of companies and enterprises; 5) professional, scientific, and technical services; 6) motor vehicle, body, trailer, and part manufacturing; 7) securities commodity contracts and investments; 8) ambulatory health care services; 9) air transportation; and 10) amusements, gambling, and recreation.

**Fig. 4.**
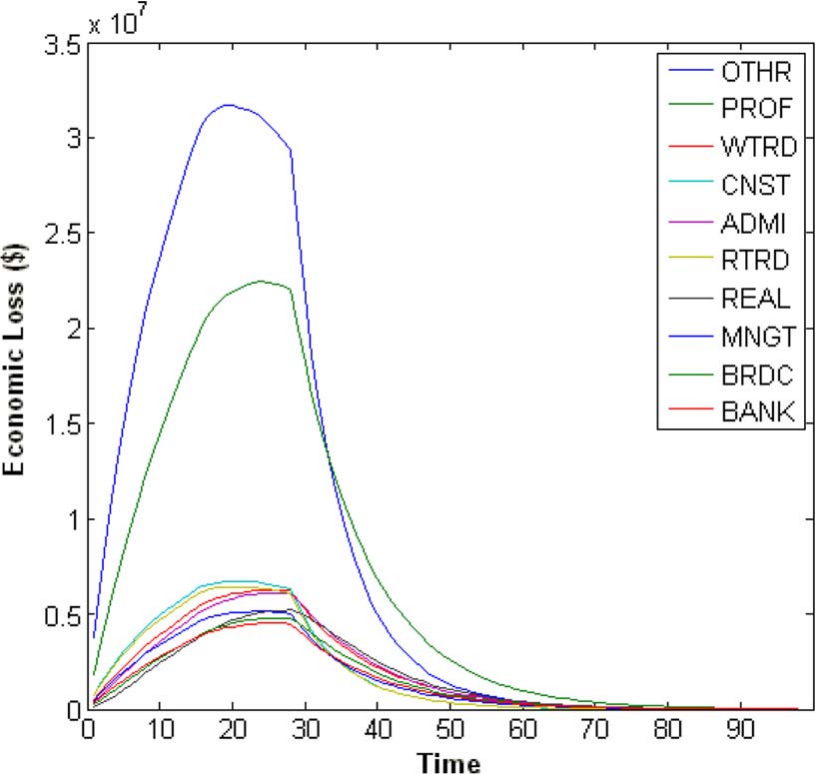
Top ten sectors with highest economic losses.

The summary of the economic losses of 59 sectors is shown in Fig. 4. In a fairly sharp contrast to the plot of inoperability, there are two sectors that clearly stand out among the rest. These two sectors are again the other services and the professional services sectors. Looking at the top ten sectors in terms of economic loss (Fig. 4), these two sectors clearly dominate the others in terms of economic loss. The other sectors which appear in the top ten are, in order, 3) wholesale trade, 4) construction, 5) administrative and support services, 6) retail trade, 7) real estate, 8) management of companies and enterprises, 9) broadcasting and telecommunications, and 10) federal reserve banks, credit intermediation, and related services.

Only three sectors appeared in the top ten for both economic loss and inoperability. They were other services, professional services, and management of companies and enterprises.

Overall, the four-week pandemic with 15% attack rate had an expected economic loss for all sectors of $4.6 billion (1.3% of GDP) with a minimum of $3.9 billion (1.1% of GDP) and a maximum of $5.5 billion (1.6% of GDP). The conditional expected value $(f_{4})$ was $5.2 billion (1.5% of GDP). It took 91 days (63 days after the pandemic had ended) for every sector to recover to 1% of its maximum inoperability.

Scenario 2: **Four-Week Pandemic, 25% Attack Rate**

The second scenario looks at the impact of a four-week pandemic with a 25% attack rate. Again, these inputs determine the number of inoperable workforce, as outputted by the FluWorkLoss model.

The major difference between the 25% attack rate and the 15% attack rate is the magnitude of the inoperability levels. While the 15% attack rate peaked around 14% inoperability, the 25% attack rate peaks around 24% inoperability for the highest sector. This means that, for an increase of 10% attack rate, we see an increase of 9% in the peak inoperability.

In terms of overall economic impact, the expected economic impact for all sectors is $7.7 billion (2.2% of GDP), the maximum was $9.1 billion (2.6% of GDP), the minimum was $6.7 billion (1.9% of GDP), and the conditional expected value $(f_{4})$ was $8.8 billion (2.5% of GDP).

Scenario 3: **Four-Week Pandemic, 35% Attack Rate**

A similar trend that occurred in Scenario 2 also applies to Scenario 3. The shapes of all plots are the same, and the relative impact to the sectors is the same as well. The peak inoperability of all sectors is 34%. A summary of the economic impact of the third scenario, along with comparison data from the previous two scenarios, is shown in Table I.

**Table I:** Economic Impact of the Three Scenarios of the Case Study

Total Economic Impact (Billions of Dollars)			
	**Scenario I**	**Scenario 2**	**Scenario 3**
**Minimum**	**3.9**	**6.6**	**9.0**
**Mean**	**4.6**	**7.7**	**10.8**
**f4**	**5.3**	**8.8**	**12.3**
**Maximum**	**5.5**	**9.1**	**12.9**

## V. Discussion and Application to Risk Management

This paper attempts to contribute to the field of disaster consequence modeling. A unique contribution of the current paper is incorporating probabilistic and temporal extensions to the well-established economic I-O model. In particular, we explicitly incorporate recovery and uncertainty bounds for estimating the adverse economic effects resulting from a workforce debilitating disaster, such as a pandemic. Economic loss estimates provide policymaking insights (e.g., evaluating feasible mitigation strategies via cost–benefit analyses). The remainder of this section provides discussions on the interplay between sector-specific consequences and risk-management policymaking.

The dominance of the other services and professional services sectors in terms of overall economic loss leads to some questions such as why those two are the most heavily impacted and what are the implications of this in terms of risk management. To address why those two sectors have far and above the greatest economic losses (despite not having such a drastically high relative level of inoperability), it is useful to look back at the input data.

A big piece of the puzzle is the relative size of these sectors, particularly in terms of GDP. The other services sector, which, in the sector classification system used here, includes all of the federal employees, accounts for nearly 20% of the GDP of the Virginia economy. Likewise, the professional services sector accounts for 11% of the total GDP. This explains why these two sectors would have such large economic losses relative to the other sectors; they are a larger scale than the other sectors.

However, these are not the two largest sectors. The real estate sector accounts for 12% of the GDP. The question then becomes why this sector is not as heavily impacted as the other two. The answer is found in the impact of the workforce on these sectors as follows. The other services sector has an 81% reliance on workforce, and the professional services sector has a 46% reliance on workforce. Real estate, on the other hand, only has a 4% reliance on workforce.

This realization has a significant impact on formulating risk-management strategies. On the one hand, it would appear that these sectors should be prioritized to minimize the economic impact of the economy. However, upon closer inspection, this recommendation should be carefully assessed. One of the major reasons why these sectors are so heavily impacted is their relative size of output; this would tend to indicate that these sectors should receive no special treatment. Additionally, many of the sectors have reliance on workforce values that are similar or higher than these two sectors. Thus, this paper provides just one of the pieces in determining how to prioritize the protection of sectors. Many other factors need to be considered as well; for example, the health and hospital sectors will probably play a very large role in containing the outbreak and minimizing the number of infections and death rate. All of these factors need to be considered by the decision makers when coming up with risk-mitigation techniques.

One of the reasons why a constant pandemic duration was chosen for the case study was that, according to discussions with the Director of Emergency Preparedness at the University of Virginia [20], intervention measures may have an impact on the attack rate or number of cases, but typically, the duration of the pandemic is inherent to the characteristics of the particular strain of the disease, and barring quarantine of individuals or rapid and widespread deployment of a highly effective vaccine, there is little that can be done to stop the pandemic from running its course in terms of the time that it will take.

However, this paper can potentially show the value of risk-management strategies as it relates the ability to mitigate the attack rate of the pandemic. In the pandemic scenarios shown in Table I, reducing the attack rate by 10% reduces the overall economic impact by about $2.8 billion. In the worst case scenarios, the savings could be in the $3–3.6 billion range. These values could be used to set some breakeven points on the value of risk management as they relate to loss-reduction opportunities.

The outcomes of these results, while hard to verify due to a lack of sufficient historical data, mesh very closely with the results obtained by a study done by the Congressional Budget Office, estimating that a pandemic would create a supply side reduction in GDP of about 2.25% [21]. The expected values of this research, i.e., 1.2%, 2.0%, and 2.8%, for scenarios 1, 2, and 3, respectively, encompass these results very closely.

Since a key contribution of this paper is on the development of a pandemic software (i.e., the MatLab structure shown in Fig. 1), future enhancements to the current version can benefit from related efforts on disaster-decision-support-tool developments, such as those pursued in [22] and [23].

## Supplementary Material

Color versions of one or more of the figures in this paper are available online at http://ieeexplore.ieee.org.
